# Artificial Intelligence and Technology for Aging: Insights From the Johns Hopkins AITC

**DOI:** 10.1093/geroni/igaf122.1523

**Published:** 2025-12-31

**Authors:** Peter Abadir

**Affiliations:** Johns Hopkins University School of Medicine & Wisting School of Engineering, Baltimore, Maryland, United States

## Abstract

The National Institute on Aging (NIA) funds the Johns Hopkins Artificial Intelligence and Technology Collaboratory for Aging Research (Hopkins AITC) to develop engineering innovations, technologies, and devices aimed at increasing independence in older adults. The Hopkins AITC is dedicated to bridging AI and biomedical advancements to support and enhance physical and cognitive health, ensuring that emerging technologies translate into practical, scalable solutions for aging populations. An important focus of the Hopkins AITC is the biological basis of physical and cognitive decline in older adults. Many of its pilot projects explore the integration of AI and novel technologies to assess, monitor, and intervene in age-related deterioration. These projects leverage wearable technology, smart devices, remote patient monitoring, and AI-driven analytics to provide insights into mobility, sleep, frailty, and cognitive function. Additionally, the Hopkins AITC has supported pilot projects focusing on caregivers and training the geriatrics workforce. These efforts include developing home monitoring devices to enhance caregiving, AI-driven tools to assist with care management, and technologies targeting social isolation in older adults. By addressing both direct patient needs and caregiver support, the Hopkins AITC aims to create a comprehensive framework for aging care. This session in the symposium will highlight key Hopkins AITC pilot projects and their contributions to aging research. Discussions will address the challenges of translating AI and engineering innovations into clinical and home settings, overcoming barriers to adoption, and ensuring ethical AI implementation.

